# A low-threshold sleep intervention for improving sleep quality and well-being

**DOI:** 10.3389/fpsyt.2023.1117645

**Published:** 2023-02-23

**Authors:** Esther-Sevil Eigl, Laura Krystin Urban-Ferreira, Manuel Schabus

**Affiliations:** ^1^Laboratory for Sleep, Cognition, and Consciousness Research, Department of Psychology, University of Salzburg, Salzburg, Austria; ^2^Centre for Cognitive Neuroscience Salzburg (CCNS), University of Salzburg, Salzburg, Austria

**Keywords:** sleep education, low-threshold, sleep hygiene, sleep coaching, actigraphy, sleep quality, sleep disorders, sleep intervention

## Abstract

**Background:**

Approximately one-third of the healthy population suffer from sleep problems, but only a small proportion of those affected receive professional help. Therefore, there is an urgent need for easily accessible, affordable, and efficacious sleep interventions.

**Objective:**

A randomized controlled study was conducted to investigate the efficacy of a low-threshold sleep intervention consisting of either (i) sleep data feedback plus sleep education or (ii) sleep data feedback alone in comparison with (iii) no intervention.

**Material and methods:**

A total of 100 employees of the University of Salzburg (age: 39.51 ± 11.43 years, range: 22–62 years) were randomly assigned to one of the three groups. During the 2-week study period, objective sleep parameters were assessed *via* actigraphy. In addition, an online questionnaire and a daily digital diary were used to record subjective sleep parameters, work-related factors, as well as mood and well-being. After 1 week, a personal appointment was conducted with participants of both experimental group 1 (EG1) and experimental group 2 (EG2). While the EG2 only received feedback about their sleep data from week 1, the EG1 additionally received a 45-min sleep education intervention containing sleep hygiene rules and recommendations regarding stimulus control. A waiting-list control group (CG) did not receive any feedback until the end of the study.

**Results:**

Results indicate positive effects on sleep and well-being following sleep monitoring over the course of 2 weeks and minimal intervention with a single in-person appointment including sleep data feedback. Improvements are seen in sleep quality, mood, vitality, and actigraphy-measured sleep efficiency (SE; EG1), as well as in well-being and sleep onset latency (SOL) in EG2. The inactive CG did not improve in any parameter.

**Conclusion:**

Results suggest small and beneficial effects on sleep and well-being in people being continuously monitored and receiving (actigraphy-based) sleep feedback when paired with a single-time personal intervention.

## Introduction

A growing number of stress-inducing factors in our fast-moving society have led to an alarming increase in the impairment of sleep quality in the general population. Especially in stressful times, sleep is often reduced [e.g., (1, 2)], while its impact on vital functions such as recovery or cognition is largely underestimated. This lack of awareness of the importance of sleep and its significant impact on mental (3) and physical health (4) is worrisome, as many people in the western world suffer from insufficient sleep, irregular sleep patterns, and/or non-restorative sleep episodes (5).

The strong association between personal worry, human sleep, and mental health factors became evident especially during the COVID-19 pandemic [e.g., (6, 7)]. Alarming adverse effects on well-being and mental health, such as the increase in depressive symptoms, anxiety, and stress (7, 8) as well as increasing sleep problems associated with decreased sleep quality (6, 7) and insomnia (9), have consequently been reported. Studies conducted during the COVID-19 pandemic show numbers reaching from 16% of the Austrian population reporting clinical or severe insomnia symptoms (9) to 37% in a huge international comparison (7) and up to nearly 60% affected by poor sleep quality in an Italian study (10). Frequent reasons for sleep problems are high work demands and associated chronic stress in the work as well as private environment (11).

Interestingly, many sleep problems remain clinically undiagnosed or untreated and consequently often manifest themselves in chronic sleep disorders (12, 13). Thus, we believe that it is inevitable to broaden access to sleep-promoting, low-threshold interventions for the general population, especially in the very early stages of developing sleep problems.

Providing information about sleep and good sleep hygiene is one of the core components of insomnia treatment (14, 15). Yet only a few studies have investigated the use of low-threshold interventions focusing on sleep education in the general population together with objective measures for a more reliable sleep evaluation using actigraphy or the gold standard of polysomnography (PSG) in comparison with a waiting-list control group (CG) (16, 17).

Indeed, previous research suggested that brief intervention programs with ≤ 4 sessions of cognitive behavioral therapy for insomnia (CBT-I) can be effective in the improvement of both subjective sleep data and actigraphy-measured sleep, especially in the long run (18). A study that explicitly compared brief behavioral treatment for insomnia (BBTI), which consists of fewer sessions and a shorter duration than classical CBT-I programs, found promising effects of BBTI on insomnia severity and improvements of sleep onset latency (SOL), sleep efficiency (SE), total wake time, wake after sleep onset (WASO), and sleep quality measured *via* sleep diaries (19); however, the study could not conclusively demonstrate non-inferiority of BBTI against the CBT-I standard.

Holzinger et al. (20) reported positive effects of a low-threshold non-pharmacological sleep intervention in the form of a 2-day sleep coaching seminar for Austrian shift workers on subjective measures of sleep quality, SOL, and daytime sleepiness. Using 1-day workshops based on the core principles of CBT-I, one such study trial found improvements in insomnia severity, subjective SE, and WASO 3 months after the baseline measurement when compared to a waiting-list CG (21). Recently, Wong et al. (22) compared two low-intensity interventions for insomnia (half-day CBT-I workshop and self-help Internet-delivered CBT-I) with an active-treatment CG (half-day sleep hygiene education workshop) and found comparable improvements in insomnia severity, anxiety, depressive symptoms, and quality of life in all three groups. Effects were stable until 8 and 16-week post-baseline assessments. In addition, a pilot study in a sample of working women with sleep disorders that included 30-min weekly group sleep hygiene education classes over a period of 5 weeks revealed significant improvements in Pittsburgh Sleep Quality Index values [PSQI; (23)] already after 2 weeks of participation (24). There are even reports that a single-time 1-h sleep hygiene session in a Japanese working population leads to decreased PSQI scores and a significantly reduced subjective daytime sleepiness 4 weeks after the intervention (25); however, like most of the trials, this study also (i) did not find significant differences compared to a waiting-list group and (ii) did not use objective measures for assessing the participants' sleep, which, therefore, is prone to placebo or “socially desired” effects. Only a few studies have met those requirements.

A 4-week program with weekly 45-min CBT-I sessions in elderly people, also including sleep-hygiene education, revealed significant improvements in (actigraphy-measured) SE and the number (and duration) of awakenings compared to an active CG; this effect was stable up to 4 months after the completion of treatment (26). Furthermore, in a sample of older adults with chronic insomnia and common comorbidities, BBTI was delivered in one 45–60-min individual session and two 20-min telephone calls and was compared with an active control condition 4 weeks after treatment and at a 6-month follow-up. Besides subjective sleep amelioration, actigraphy-based improvements in SE, SOL, and WASO were also observed. However, in polysomnographic parameters, no treatment effects could be determined (16). In contrast, Krystal et al. (27) found a CBT-I group compared with a placebo intervention group with 6 weekly sessions each to show greater improvements in ambulatory PSG measures of WASO and SE from before to after the treatment.

Digital intervention solutions gain importance year by year (28, 29) and got a boost in the time of the COVID-19 pandemic. Often, however, these programs cannot be classified as “low-threshold,” as the duration is rather long (6–8 weeks) and intensity is high (weekly 60-min meetings). Among the shorter versions and, therefore, being more low level, a 2-week online program based on cognitive-behavioral intervention could improve subjective sleep quality and subjective SOL among adult workers when compared to a waiting-list CG (30). In a large comparison of digital CBT-I with sleep hygiene education, improvements in sleep-related quality of life, insomnia, and psychological well-being could be shown already at mid-treatment (week 4) (31). Using an individually tailored app-based treatment for insomnia for 2 weeks compared to a waiting-list CG, Okajima et al. (32) found the treatment group to be more improved in insomnia severity both at a 1- and 3-month follow-up. Yet, here as well, reports fully rely on subjective reports, and there is a lack of sleep and treatment evaluation using objective sleep measures.

In summary, it becomes evident that an overwhelming amount of existing sleep studies using low-threshold sleep interventions rely on subjective measures of sleep [e.g., (20–22, 33)] and, thereby, have inherent problems arising from possibly biased self-reports. This is especially problematic regarding sleep, which by definition is a state of “unconsciousness” [e.g., (34)]. In addition to this, many studies lack a proper CG that evaluates whether simple personal contact and focus on one's own sleep leads to (subjective) sleep improvements independent of specific treatment effects. A possible and economical way to enhance the objectivity of measuring sleep in intervention studies is the use of actigraphy (35) or other reliable sensor data. Although actigraphy cannot substitute the established gold standard of PSG, some studies suggest that it still provides acceptable accuracy for many sleep parameters (36), especially if data are collected for at least six to seven continuous nights (37).

In the current study, we apply a single-time face-to-face sleep intervention in a sample of 100 employees of an Austrian University (University of Salzburg). We quantify improvements in sleep using daily objective sleep parameters gained from wrist-worn actigraphy and daily subjective sleep parameters over the course of 2 weeks.

## Materials and methods

### Sample

The sample comprised 100 volunteers of the working staff of the University of Salzburg, of whom 69% (*n* = 69) were women. Their age ranged between 22 and 62 years (*M* = 39.51 ± 11.43). The majority worked in science (46%), followed by 26% who were working in administration. Besides this, 15% had teaching assignments, and 35% worked in “other” sectors of the university. Regarding the educational qualification, 72% of the participants had a university degree (*n* = 72), 16% had graduated from high school (*n* = 16), and the remaining 12% had undergone educational training (*n* = 7), engaged in further education (*n* = 3), or attended primary or secondary school (*n* = 2). Most of the participants had full-time jobs (35+ h, *n* = 47) or were engaged in part-time work (15–35 h a week, *n* = 41). The remaining were employed for less than 15 h a week (*n* = 10) or declared that they had two jobs or were just searching for new employment (*n* = 2).

After confirmation of participation, the participants were randomly assigned to one of the three groups [i.e., experimental group 1 (EG1), experimental group 2 (EG2), and CG], while the time point of confirmation was the determining factor for the alternating group allocation to circumvent time effects. There were no exclusion criteria other than that the participants had to be members of the (scientific or non-scientific) working staff of the University of Salzburg, and sleep improvement was desired.

### Objective measurement

Objective sleep data were measured by using ©wGT3X-BT ActiGraphs (ActiGraph™; Pensacola, FL, USA), which capture continuous, high-resolution physical activity and provide sleep/wake analysis. ActiGraph data were processed using the ©ActiLife software and the implemented Cole–Kripke algorithm (38) with a pre-defined sleep epoch length of 60 s; we here focused on SE and SOL of that algorithm. In addition, participants were asked to keep a daily sleep log, where individual lights on/off was recorded. Participants were instructed to provide these timings immediately before switching off and after turning on the lights.

### Subjective measurement

A few days before the start of the pre-intervention period, participants filled out an entry questionnaire (T1), where demographic data and information about individual sleep problems using the PSQI were assessed. The PSQI reliably measures the self-reported sleep quality over the past 4 weeks by means of 19 items and covers seven areas, namely, subjective sleep quality, sleep latency, sleep duration, habitual SE, sleep disturbances, use of sleep medication, and daytime dysfunction. The global PSQI score ranges from 0 to 21, while a value of >5 suggests “bad sleep,” and a value of >10 is considered a sleep disorder with clinical relevance. Furthermore, work-related demands and resources [COPSOQ; (39)], work-related distress and eustress (40), as well as subjective general well-being [HSWBS; (41)] were collected in this questionnaire. The COPSOQ, work-related distress and eustress, and the HSWBS were collected again at T2 and T3^1^. In addition to this, participants completed daily morning and evening protocols, which were available using the “PsyDiary” mobile-phone app (Eating Behavior Laboratory, University Salzburg–SmartHealthCheck Project). The morning diary (refer to Supplementary Table 1) collected (based on one item each) the subjective evaluation of the previous night's sleep quality (“How did you sleep tonight?”, from “very bad” to “very good”), self-reported vitality (“How do you feel at the moment?”, from “faint” to “alive”), and current mood (“How is your mood at the moment?”, from “very bad” to “very good”) *via* a slider bar from 0 to 100. Furthermore, subjective psychological well-being was assessed as part of the morning diary by the use of an adapted version of the WHO-Five Well-Being Index (42), which is answered on a 5-point Likert scale and consists of five statements like, for example, “I feel cheerful and in good spirits.” In the evening, the evaluation of daily job demands and resources, self-reported tension, psychological detachment, and daily sleepiness was assessed using 15 questions (refer to Supplementary Table 2). Participants were instructed to fill out the daily protocol in the morning 30–60 min after turning on the lights and in the evening at least 2 h before going to bed. Questionnaire data (i.e., T2 and T3) were obtained before the personal meetings with the study team.

### Sleep interventions

**Figure 1:**
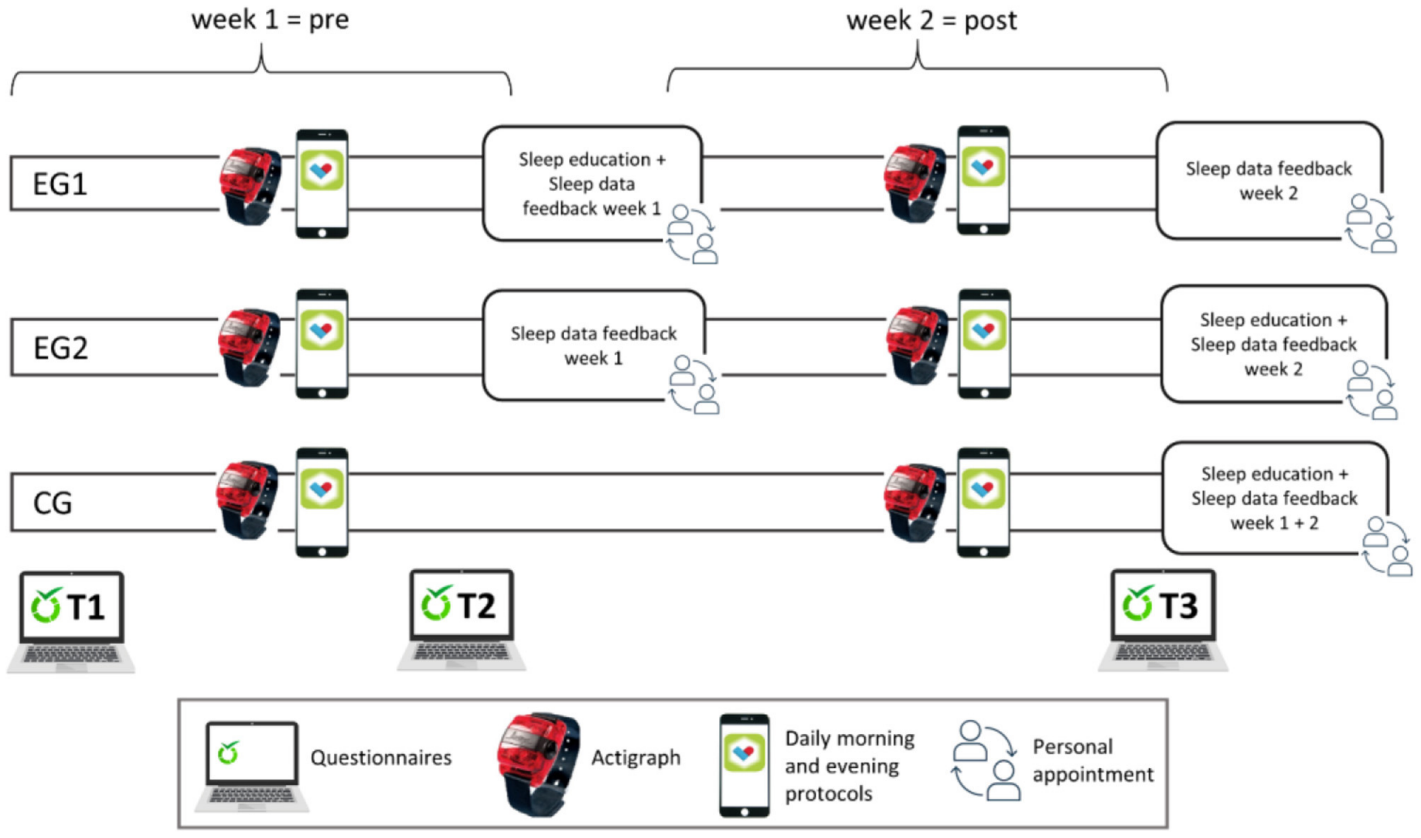
Study design. Participants wore an actigraph for 2 weeks and filled out questionnaires at three points of time as well as two daily protocols (morning and evening) *via* a mobile phone app (PsyDiary). During a personal appointment (after week 1), experimental group 1 (EG1) received feedback on their sleep data from the first week plus a sleep education intervention, while EG2 received sleep data feedback only. The waiting-list control group (CG) was monitored like the other groups, but only had a personal appointment and got feedback after the end of the 2-week study period.

At the midpoint of the study period (after 1 week), the EG1 received a face-to-face, 45–60-min lasting session with a member of the Laboratory for Sleep, Cognition and Consciousness Research of the University of Salzburg, who was trained for the “sleep education intervention.” General information about sleep, basic principles of sleep hygiene, as well as general advice for better sleep (stimulus control), were explained and discussed by the use of an information booklet. In addition, the “coach” showed and explained the participants' sleep data of the first week as recorded from the actigraph. Furthermore, the sleep data feedback addressed the PSQI value at study entrance. The EG2 received sleep data feedback only, at the same point of time in the study protocol (namely 1 week after study entrance). Finally, the CG was simply monitored for the 2-week period with actigraphy and daily morning and evening protocols *via* smartphone app and only had a personal appointment to discuss the data at the end of the study period (cf. Figure 1).

### Statistical analysis

Statistical analyses were performed using SPSS version 24 (SPSS Inc., Chicago, Illinois). To test whether the data were normally distributed, Shapiro–Wilk tests were used. Analyses were mainly based on repeated measure analyses of variance (ANOVA), *t*-tests, or Wilcoxon tests, depending on the distribution of the data. As suggested by Wasserstein et al. (43), we interpreted the overall pattern rather than focusing on individual *p*-values. Therefore, we also interpreted *p*-values of 0.05 < *p* ≤ 0.1 as statistical trends, if they were in line with the overall pattern. For a measure of effect size, either partial eta squared (η^2^*p*) or the correlation coefficient (*r*) are provided, depending on the inferential statistics applied. The Greenhouse–Geisser correction was used in case the assumption of sphericity was violated. For *post-hoc* comparisons, parametric tests (*t*-tests) or Wilcoxon-rank-sum-tests were applied (depending on the distribution of the data), and two-tailed critical *p*-values were reported. There were no significant differences in the investigated outcome variables between the groups at time point T1 (pre). For descriptive values of the outcome variables at time point T1, refer to Supplementary Table 3. Pre-measurements refer to the mean of the first week (T1 to T2) and post-measurements refer to the mean of the second week (T2 to T3) of continuous morning diary and/or actigraphy data. As in the EG2, there were two participants who did not fill out the sleep diary during the pre-intervention period, only the data of *n* = 32 participants could be included in the analyses of subjective data for the EG2.

## Results

### Sleep diary

#### Sleep quality

**Figure 2:**
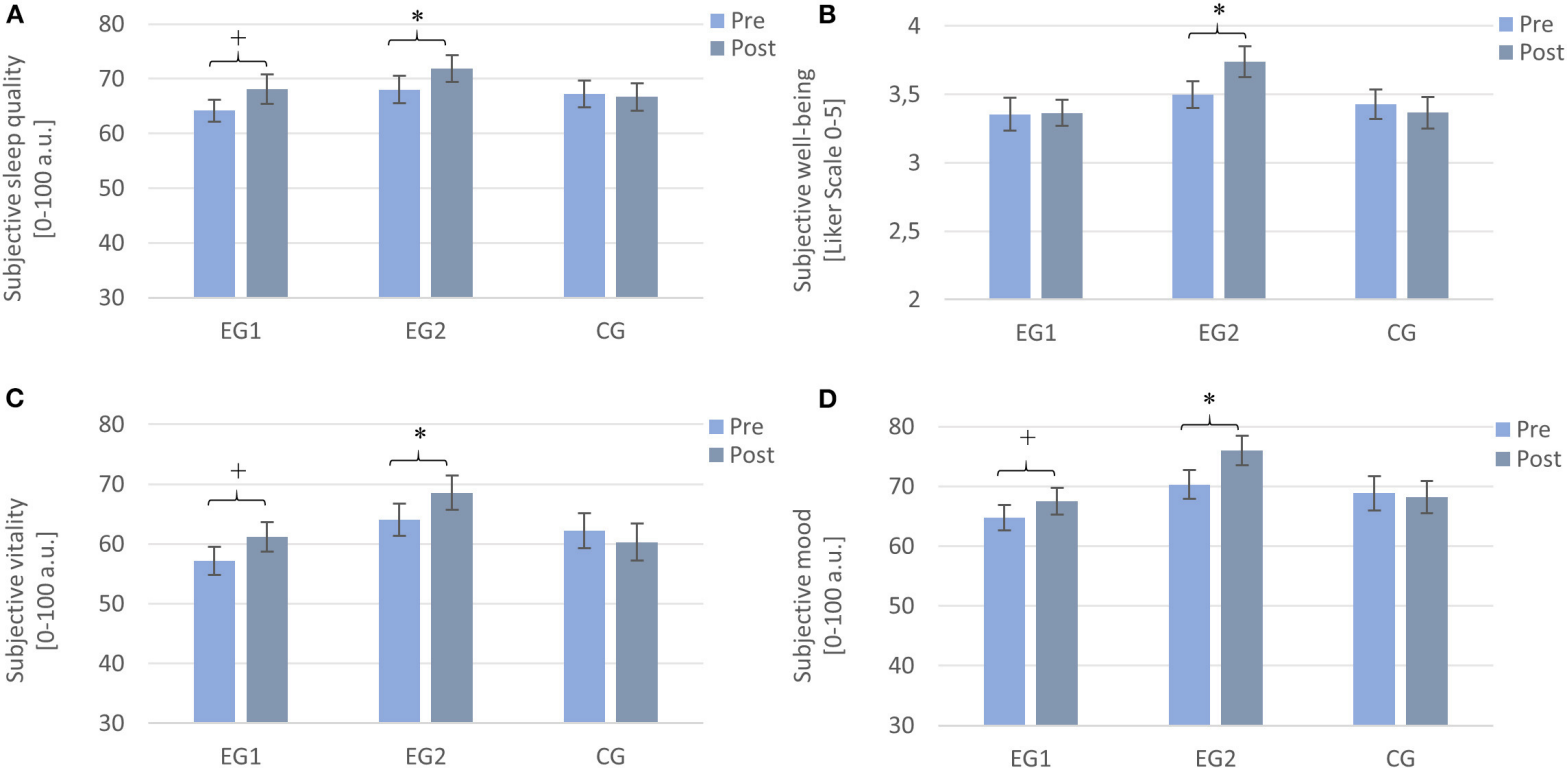
Subjective values assessed by sleep diaries from pre- to post-intervention. Note that EG1 improved tendentially in **(A)** sleep quality, **(C)** vitality, and **(D)** mood, but not in **(B)** well-being, and the EG2 improved significantly on all parameters, while the CG did not show any improvements. Measurements were assessed daily and averaged over a week each. EG1 (*n* = 34), EG2 (*n* = 32), and CG2 (*n* = 32). Asterisks represent significant results, *p < 0.05, +*p* < 0.10, two-tailed. Error bars display ±1 standard error. EG1, experimental group one; EG2, experimental group two; CG, control group.

A 2 × 3 repeated measures ANOVA with the within-subject factor TIME (Pre and Post), the between-subject factor GROUP (EG1, EG2, and CG), and the dependent variable subjective sleep quality revealed a significant main effect of TIME [*F* (1, 95) = 4.70, *p* = 0.033, η^2^*p* = 0.047], indicating that subjective sleep quality increased significantly over participants from pre- (*M* = 66.44, *SD* = 13.35) to post-intervention (*M* = 68.87, *SD* = 14.48). The main effect of GROUP was not significant [*F* (2, 95) = 0.80, *p* = 0.451, η^2^*p* = 0.017] nor was the interaction TIME × GROUP [*F* (2, 95) = 1.77, *p* = 0.176, η^2^*p* = 0.036]. Explorative *post hoc* paired samples *t*-tests revealed a trend for a significant increase in subjective sleep quality from pre to post for the EG1 [Pre: *M* = 64.17 ± 11.92, Post: *M* = 68.07 ± 15.54; *t*(33) = −1.96, *p* = 0.058, *r* = 0.32] and a significant increase for the EG2 [Pre: *M* = 68.03 ± 14.34, Post: *M* = 71.87 ± 13.59; *t* (31) = −2.31, *p* = 0.028, *r* = 0.38] but no change for the CG [Pre: *M* = 67.26 ± 13.84, Post: *M* = 66.70 ± 14.11; *t* (31) = 0.27, *p* = 0.787] (cf. Figure 2A).

#### Well-being

A 2 × 3 repeated measures ANOVA with the within-subject factor TIME (Pre and Post), the between-subject factor GROUP (EG1, EG2, and CG), and the dependent variable well-being revealed no significant main effect of TIME [*F* (1, 95) = 0.19, *p* = 0.274, η^2^*p* = 0.013] and no significant main effect of GROUP [*F* (2, 95) = 2.04, *p* = 0.136, η^2^*p* = 0.041]. There was a trend for a significant interaction effect TIME × GROUP [*F* (2, 95) = 2.56, *p* = 0.083, η^2^*p* = 0.051]. Subsequent *post-hoc* Wilcoxon tests revealed a significant increase in well-being from pre to post only for the EG2 (Pre: *M* = 3.49 ± 0.56, Post: *M* = 3.74 ± 0.64; *Z* = –2.50, *p* = 0.013, *r* = 0.44). For the EG1 (Pre: *M* = 3.35 ± 0.70, Post: *M* = 3.37 ± 0.56; *Z* = –1.282, *p* = 0.200) and the CG (Pre: *M* = 3.43 ± 0.61, Post: *M* = 3.37 ± 0.66; *Z* = –0.442, *p* = 0.658), there was no significant change from pre- to post-measurement (cf. Figure 2B).

#### Vitality

A 2 × 3 repeated measures ANOVA with the within-subject factor TIME (Pre and Post), the between-subject factor GROUP (EG1, EG2, and CG), and the dependent variable vitality revealed a trend toward a significant main effect of TIME [*F* (1, 95)= 3.04, *p* = 0.084, η^2^*p* = 0.031] while the main effect of GROUP was not significant [*F* (2, 95) = 2.13, *p* = 0.125, η^2^*p* =0.043]. For the interaction effect TIME × GROUP, there was a trend toward a significant effect [*F* (2, 95) = 2.66, *p* = 0.075, η^2^*p* =0.053]. *Post-hoc* Wilcoxon tests revealed a trend for an increase in the feeling of vitality for the EG1 (Pre: *M* = 57.18 ± 13.57, Post: *M* = 61.21 ± 14.47; *Z* = −1.85, *p* = 0.065, *r* = 0.32) and a significant increase for the EG2 (Pre: *M* = 64.08 ± 15.40, Post: *M* = 66.99 ± 18.19; *Z* = −2.23, *p* = 0.026, *r* = 0.39) but not for the CG (Pre: *M* = 62.24 ± 16.57, Post: *M* = 60.31 ± 17.62; *Z* = −0.44, *p* = 0.660) (cf. Figure 2C).

#### Mood

A 2 × 3 repeated measures ANOVA with the within-subject factor TIME (Pre and Post), the between-subject factor GROUP (EG1, EG2, and CG), and the dependent variable mood revealed a significant main effect of TIME [*F* (1, 95) = 7.90, *p* = 0.006, η^2^*p* = 0.077], while the main effect of GROUP was not significant [*F* (2, 95) = 2.33, *p* = 0.103, η^2^*p* = 0.047]. The interaction effect TIME × GROUP was significant [*F* (2, 95) = 3.89, *p* = 0.024, η^2^*p* = 0.076]. *Post-hoc* paired samples *t*-tests revealed a trend toward an increase in mood for the EG1 [Pre: *M* = 64.74 ± 12.54, Post: *M* = 67.49 ± 13.15; *t* (33) = −1.89, *p* = 0.068, *r* = 0.31] and a significant improvement for the EG2 [Pre: *M* = 70.32 ± 13.72, Post: *M* = 75.97 ± 13.84; *t* (31) = −3.50, *p* = 0.001, *r* = 0.53] but no change for the CG [Pre: *M* = 68.86 ± 16.07, Post: *M* = 68.19 ± 15.45; *t* (31) = 0.40, *p* = 0.694] (cf. Figure 2D).

### Actigraphy

#### Sleep efficiency

**Figure 3:**
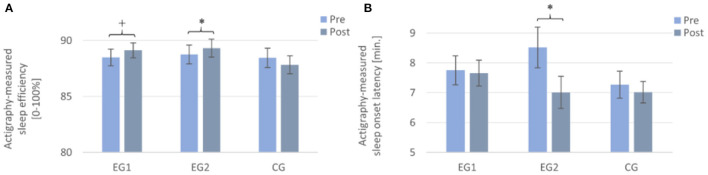
Actigraphy-recorded parameters from pre to post in all three groups. Note that the EG1 showed a trend toward an improvement from pre to post in **(A)** sleep efficiency, but not in **(B)** sleep onset latency; the EG2 improved significantly from pre to post in both measures, while the CG showed no improvement. Measurements were assessed daily and averaged over a week each. EG1 (*n* = 34), EG2 (*n* = 34), and CG (*n* = 32). Asterisks represent significant results, *p < 0.05, +*p* < 0.10, two-tailed. Error bars display ±1 standard error. EG1, experimental group one; EG2, experimental group two; CG, control group.

A 2 × 3 repeated measures ANOVA with the within-subject factor TIME (Pre and Post), the between-subject factor GROUP (EG1, EG2, and CG), and the dependent variable (actigraphy-measured) SE revealed no significant result for the main effect of TIME [*F* (1, 97) = 0.89, *p* = 0.349, η^2^*p* = 0.009] and of GROUP [*F* (2, 97) = 0.44, *p* = 0.643, η^2^*p* = 0.009], but a significant interaction effect of TIME × GROUP [*F* (2, 97) = 3.32, *p* = 0.040, η^2^*p* = 0.064] and, therefore, confirmed the above effects. Subsequent *post-hoc* paired samples *t*-tests revealed a trend toward increased objective SE from pre to post for the EG1 [Pre: *M* = 88.50 ± 4.33, Post: *M* = 89.12 ± 3.90; *t* (33) = −1.78, *p* = 0.085 *r* = 0.25] and a significant increase for the EG2 [Pre: *M* = 88.80 ± 4.67, Post: *M* = 89.46 ± 4.51; *t* (33) = −2.14, *p* = 0.040, *r* = 0.35]. For the CG, the paired samples *t*-test showed no significant difference [Pre: *M* = 88.46 ± 4.89, Post: *M* = 87.83 ± 4.55; *t* (31) = 1.21, *p* = 0.236] (cf. Figure 3A).

#### Sleep onset latency

A 2 × 3 repeated measures ANOVA with the within-subject factor TIME (Pre and Post), the between-subject factor GROUP (EG1, EG2, and CG), and the dependent variable (actigraphy-measured) SOL revealed a significant result for the main effect of TIME [*F* (1, 97) = 4.65, *p* = 0.034, η^2^*p* = 0.046] and a trend toward a significant interaction effect of TIME × GROUP [*F* (2, 97) = 2.61, *p* = 0.078, η^2^*p* = 0.051]. The main effect for GROUP [*F* (2, 97) = 0.87, *p* = 0.424, η^2^*p* = 0.018] was not significant. Subsequent *post-hoc* paired samples *t*-tests revealed a significant decrease of objective SOL only for the EG2 [Pre: *M* = 8.73 ± 3.90, Post: *M* = 7.01 ± 2.96; *t* (33) = 2.7, *p* = 0.011, *r* = 0.43]. For the EG1 [Pre: *M* = 7.75 ± 2.86, Post: *M* = 7.66 ± 2.53; *t* (33) = 0.16, *p* = 0.872] and the CG [Pre: *M* = 7.27 ± 2.54, Post: *M* = 7.02 ± 2.06; *t* (31) = 0.69, *p* = 0.493], the paired samples *t*-test showed no significant difference from pre- to post-measurement (cf. Figure 3B).

### Sleep intervention vs. control

As the EG1 and the EG2 both had a personal appointment after week 1 and the observed effects from pre- to post-intervention (Figures 2, 3) are of very similar direction and size, we additionally pooled EG1 and EG2 and highlight the differences between the “sleep intervention” group (*n* = 68) and the inactive CG (*n* = 32).

#### Sleep diary

##### Sleep quality

**Figure 4:**
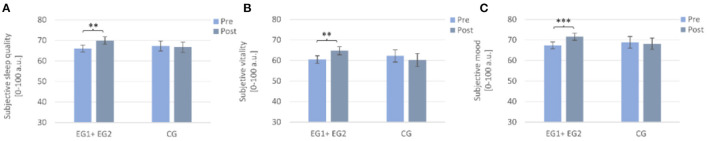
Subjectively assessed changes from pre to post in the sleep intervention group (EG1 + EG2) compared to the waiting-list control group (CG). Note that the sleep intervention group improved significantly from pre to post in **(A)** sleep quality, **(B)** subjective vitality, and **(C)** subjective mood. Pre- and post-measurements were assessed daily and averaged over a week each. Asterisks represent significant results, **p < 0.01, ***p < 0.001. Error bars display ±1 standard error. EG1+EG2, pooled sleep intervention group (experimental group one + experimental group two); CG, control group.

A 2 × 2 repeated measures ANOVA with the within-subject factor TIME (Pre and Post), the between-subject factor GROUP (EG1+EG2 and CG), and the dependent variable subjective sleep quality revealed no significant main effect for TIME [*F* (1, 96) = 2.00, *p* = 0.160, η^2^*p* = 0.020], nor for GROUP [*F* (1, 96) = 0.13, *p* = 0.720, η^2^*p* = 0.001], but a trend for an interaction effect TIME × GROUP [*F* (1, 96) = 3.58, *p* = 0.062, η^2^*p* = 0.036]. *Post-hoc t*-tests revealed significant improvements in daily subjective sleep quality from pre to post only for the “sleep intervention group” (EG1 + EG2) [Pre: *M* = 66.04 ± 13.19, Post: *M* = 69.92 ± 14.64; *t* (65) = −2.99, *p* = 0.004, *r* = 0.35] but not for the CG [Pre: *M* = 67.26 ± 13.84, Post: *M* = 66.70 ± 14.11, *t* (31) = 0.27, *p* = 0.787] (cf. Figure 4A).

##### Vitality

A 2 × 2 repeated measures ANOVA with the within-subject factor TIME (Pre and Post), the between-subject factor GROUP (EG1+EG2 and CG), and the dependent variable vitality revealed neither a significant main effect of TIME [*F* (1, 96) = 0.76, *p* = 0.387, η^2^*p* = 0.008] nor of GROUP [*F* (1, 96) = 0.19, *p* = 0.661, η^2^*p* = 0.002]. The interaction effect TIME × GROUP was significant [*F* (1, 96) = 5.35, *p* = 0.023, η^2^*p* =0.053]. *Post-hoc* Wilcoxon tests revealed a significant increase in the feeling of vitality from pre to post only for the “sleep intervention group” (EG1+EG2) (Pre: *M* = 60.52 ± 14.79, Post: *M* = 64.10 ± 16.57; *Z* = −2.95, *p* = 0.003, *r* = 0.36) but not for the CG (Pre: *M* = 62.24 ± 16.57, Post: *M* = 60.31 ± 17.62; *Z* = −0.44, *p* = 0.660) (cf. Figure 4B).

##### Mood

A 2 × 2 repeated measures ANOVA with the within-subject factor TIME (Pre and Post), the between-subject factor GROUP (EG1 + EG2 and CG), and the dependent variable mood revealed a trend toward a significant main effect of TIME [*F* (1, 96) = 3.16, *p* = 0.079, η^2^*p* = 0.032], while the main effect of GROUP was not significant [*F* (1, 96) = 0.12, *p* = 0.735, η^2^*p* = 0.001]. The interaction effect TIME × GROUP was significant [*F* (1, 96) = 6.07, *p* = 0.016, η^2^*p* =0.059]. *Post-hoc* paired samples *t*-tests revealed a significant improvement in mood from pre to post only for the “sleep intervention group” (EG1+EG2) [Pre: *M* = 67.45 ± 13.33, Post: *M* = 71.60 ± 14.05; *t* (65) = −3.81, *p* < 0.001, *r* = 0.43] but not for the CG [Pre: *M* = 68.86 ± 16.07, Post: *M* = 68.19 ± 15.45; *t* (31) = 0.40, *p* = 0.694] (cf. Figure 4C).

#### Actigraphy

##### Sleep efficiency

**Figure 5:**
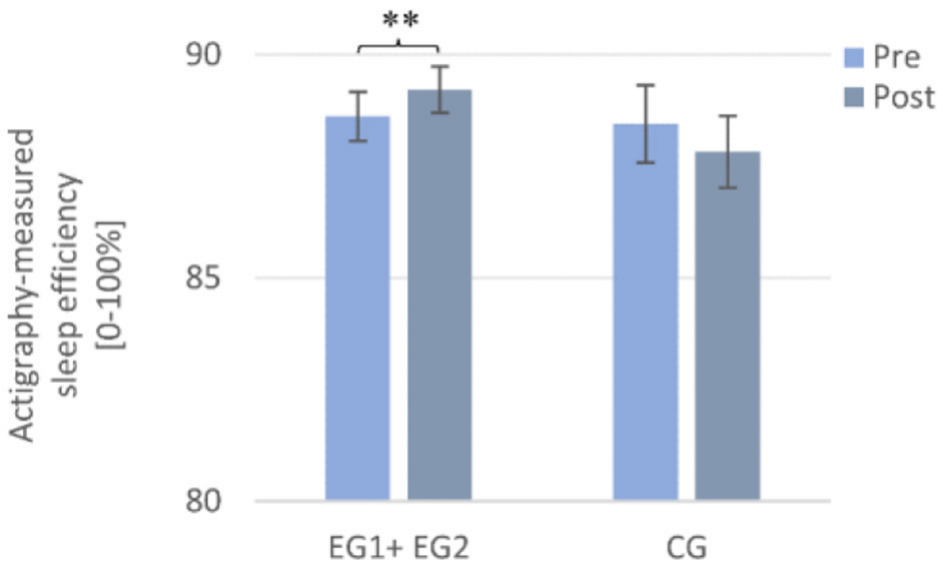
Actigraphy-recorded changes in sleep efficiency from pre to post in the sleep intervention group (EG1 + EG2) compared to the waiting-list control group (CG). Note that the sleep intervention group improved significantly from pre to post in actigraphy-measured sleep efficiency. Pre- and post-measurements were assessed daily and averaged over a week each. Asterisks represent significant results, **p < 0.01. Error bars display ±1 standard error. EG1 + EG2, pooled sleep intervention group (experimental group 1 + experimental group 2); CG, control group.

A 2 × 2 repeated measures ANOVA with the within-subject factor TIME (Pre and Post), the between-subject factor GROUP (EG1 + EG2 and CG) and the dependent variable (actigraphy-measured) SE revealed no significant main effect of TIME [*F* (1, 98) = 0.00, *p* = 0.984, η^2^*p* = 0.00] or for GROUP [*F* (1, 98) = 0.80, *p* = 0.373, η^2^*p* = 0.008] but a significant interaction effect TIME × GROUP [(*F* (1, 98) = 6.70, *p* = 0.011, η^2^*p* = 0.064]. According to *post-hoc* tests, only the “sleep intervention group” improved significantly in (actigraphy-measured) SE from pre- to post-intervention [Pre: *M* = 88.65 ± 4.47, Post: *M* = 89.29 ± 4.18; *t* (67) = −2.77, *p* =0.007, *r* = 0.32], but the CG did not [Pre: *M* = 88.46 ± 4.89, Post: *M* = 87.83 ± 4.55; *t* (31) = 1.21, *p* = 0.236] (cf. Figure 5).

## Discussion

The present study confirms that even a single, face-to-face intervention can have a positive yet small impact on the participants' sleep, well-being, and mood. Importantly, we here sampled 100 adults working at an Austrian university and not from a young student population. Surprisingly, most subjective as well as objective sleep parameters improved from pre to post with moderate effect sizes in both intervention groups (i.e., EG1 and EG2), although participants were provided with just a brief personal meeting and sleep data feedback using actigraphy over the course of 2 weeks. Sleep parameters of a waiting-list CG did not change in any of the parameters.

Concerning actigraphy-based SE, the analyses reveal (marginal) significant effects in both intervention groups. These findings are in good agreement with previous studies, which found a beneficial influence of minimal sleep hygiene interventions on actigraphy-measured parameters (16) and sleep quality in working populations and which span interventions from only a few hours (25) to 5 weekly meetings for 30 min (24).

It might appear surprising to see improvements in the EG2, although it only had one single personal feedback of ~45 min focusing on the participants' questionnaire and personal actigraphy data in the middle of the 2-week study period. Yet, most of the participants mentioned that they were pleased by the actigraphy-based sleep feedback, as their subjective estimates of sleep were usually considerably worse, and sleep feedback was perceived as a relief. This fits with data from Gavriloff et al. (44) who found that even sham feedback if positive (using actigraphy-derived SE) improves mood and alertness and can lead to a reduction of subjectively perceived sleepiness in patients with insomnia. Further sleep education of our EG1 did not seem to have an additional effect, yet we expect that catamnesis after a few months, which we, unfortunately, did not include in this study, may have revealed more stable and long-lasting effects on sleep in this group. What we could do was follow up with participants and collect the PSQI global scores of 43 participants again in fall 2022, i.e., 36 months after the study started. As it was an *ad-hoc* follow-up 3 years after the study started, it was not possible to reach all the participants. Interestingly, we here found that EG1 was the only group, which had a sustained improvement in sleep quality, manifested in significantly improved PSQI values up to date (refer to Supplementary Table 4). We speculate that administering the sleep education during the study period (EG1) was presumably more effective compared to administering it at the end of the study protocol (EG2 and CG), as participants of the EG1 had the chance to apply the rules immediately during the study period while knowing that they were still monitored. Furthermore, the EG1 was the most affected group (most participants with PSQI>10) and, therefore, might also have had the strongest adherence to the sleep education rules.

The most important factor which might have contributed to improvements in both experimental groups, is the personal contact that both groups encountered. This is in line with research, which suggests self-administered CBT-I with the support of a psychotherapist to be more effective than self-administered CBT-I alone (45, 46).

Surprisingly, improvements appeared greater from pre to post for the EG2 than for the EG1. Although the chi-square test revealed no significant difference regarding the distribution of PSQI categories (“good sleepers”/“bad sleepers”/“bad sleepers with clinical relevance”) between the groups, we believe that the less pronounced effects in the EG1 are due to the shortcoming that in the EG1, there were many more “bad sleepers with clinical relevance” than in the EG2 (PSQI > 10; EG1: *n* = 10 vs. EG2: *n* = 5) and only half as many “good sleepers” (PSQI < 5; EG1: *n* = 7 vs. EG2: *n* = 14). Indeed, exploratory analyses revealed that sleep quality improved significantly in EG1 and with a tendency in EG2 if participants with clinically disturbed sleep (PSQI > 10) were excluded from the analyses (refer to Supplementary Table 5, Figure 1).

In fact, we found that those rated as “good sleepers” (given the PSQI) seem to profit more (according to effect sizes) from the intervention than those rated as “bad sleepers.” That could be explained by the fact that PSQI-defined “bad sleepers” might be more chronic bad sleepers who cannot benefit much from such short and minimal interventions as tested here. Chronic “bad sleepers” may also have gathered information about the basic recommendations for better sleep and may, therefore, already have been familiar with the information provided in our sleep education session. This previous knowledge about sleep and sleep hygiene could influence the efficacy of the intervention and was not assessed in the present investigation.

Altogether, this suggests that minimal interventions as evaluated here should be delivered at an early stage in the general population before sleep problems become chronic.

Regarding the group differences mentioned, both minimal intervention groups were then combined and compared to the waiting-list CG. The results show that both subjectively and objectively measured sleep parameters improved from the first to the second week and correspond to findings, which demonstrated improvements in well-being and sleep behavior with the help of simple sleep hygiene interventions (20, 25). Finally, it was observed that our sleep intervention also enhanced vitality and mood from pre- to post-evaluation. This is in line with previous findings that show that high sleep quality is associated with good well-being (47) and increased positive affect (48).

As a limitation, it should be mentioned that the changes seen as a result of our sleep intervention are small in absolute numbers and, therefore, the clinical significance of such effects may be a matter of discussion. In contrast, the results of our study highlight that even a truly ‘minimal intervention' with only one session (EG1) or review of 1 week of sleep data (EG2) can evoke statistically meaningful changes in sleep parameters and well-being.

Looking at our sample from a clinical perspective, it is most alarming that almost two-thirds (67%) of our participants, who were employees of the University of Salzburg (Austria), had a PSQI score of >5, which indicates poor sleep and relevant sleep problems. This proportion is twice as high as reported for the Austrian population by Zeitlhofer et al. in 2000 (49) but almost identical to what was recently found by Blume et al. (50) with 69% poor or very poor sleepers in an online-surveyed convenience sample in the Austrian population.

A further limitation to be mentioned is that although our study had a completely randomized group allocation, we, unfortunately, got an unequal distribution of good and bad sleepers (based on PSQI) in our groups, with the EG1 having nearly 80% (PSQI-based) bad sleepers, and in contrast, EG2 and CG having about 60% bad sleepers each. However, actigraphy-derived measures indicated that the participants' objective sleep was comparable among groups with SE ranging around 89% across all groups at baseline. Although it is a clear strength of the present “low-threshold intervention” study to not only rely on subjective measures of sleep and to include 2 weeks of continuous actigraphy measurements, we are aware that this is not the accepted gold standard. In our upcoming studies, we, therefore, intend to include ambulatory data (sensors and PSG) to measure sleep and specifically the distribution of different sleep stages in the familiar home environment of the participant seeking help for better sleep.

## Conclusion

The results of this study show that a minimal intervention with actigraphy-based monitoring of sleep and personal feedback regarding individual sleep over the course of 2 weeks is promising for having positive effects on sleep as well as general well-being. The present findings indicate that face-to-face, low-threshold sleep feedback can be used at an early stage in a preventive manner in the general population and should be evaluated in more detail and extended until clinically meaningful effects are established. Here, we document that improvements in subjective and objective sleep parameters as well as in sleep-related factors are feasible with minimal time investment, which may be especially relevant in times when big proportions of the world population suffer from bad sleep, which worsened during the current coronavirus pandemic (6, 7). Ideally, future studies should, therefore, evaluate such minimal intervention programs using PSG and include catamnesis several months later. In summary, the data are promising and highlight the importance of non-pharmacological, sleep-promoting programs that are easily accessible, affordable, time-efficient, and, therefore, applicable to the general public.

## Data availability statement

The raw data supporting the conclusions of this article will be made available by the authors, without undue reservation.

## Ethics statement

The studies involving human participants were reviewed and approved by the research Ethics Committee of the Paris Lodron University of Salzburg. The patients/participants provided their written informed consent to participate in this study.

## Author contributions

E-SE, LU-F, and MS contributed to the conception and design of the study. E-SE and LU-F were responsible for data collection, performed the statistical analysis, and wrote the first draft of the manuscript. E-SE and MS revised the manuscript. MS gave final approval for the version to be published. All authors contributed to the article and approved the submitted version.

## Footnotes

^1^The PSQI was not used as an outcome measure at the end of the 2-week study period, as traditionally subjective sleep quality is evaluated over the last 4 weeks. We here rather focused on the daily measurements related to sleep quality from the online morning protocol as well as on the objective measures from the 2-week continuous sleep monitoring *via* actigraphy.

## References

[1] Yap Y Slavish DC Taylor DJ Bei B Wiley JF. Bi-directional relations between stress and self-reported and actigraphy-assessed sleep: a daily intensive longitudinal study. Sleep. (2020) 43:zsz250. 10.1093/sleep/zsz250 31608395 PMC7066487

[2] Kashani M Eliasson A Vernalis M. Perceived stress correlates with disturbed sleep: a link connecting stress and cardiovascular disease. Stress. (2012) 15:45–51. 10.3109/10253890.2011.578266 21682656

[3] Irwin MR. Why sleep is important for health: a psychoneuroimmunology perspective. Annu Rev Psychol. (2015) 66:143–72. 10.1146/annurev-psych-010213-115205 25061767 PMC4961463

[4] Besedovsky L Lange T Haak M. The sleep-immune crosstalk in health and disease. Physiol Rev. (2019) 99:1325–80. 10.1152/physrev.00010.2018 30920354 PMC6689741

[5] Chattu VK Manzar MD Kumary S Burman D Spence DW Pandi-Perumal SR. The global problem of insufficient sleep and its serious public health implications. Healthcare. (2018) 7:1–16. 10.3390/healthcare7010001 30577441 PMC6473877

[6] Florea C Topalidis P Hauser T Angerer M Kurapov A Leon CAB. Sleep during COVID-19 lockdown: a cross-cultural study investigating job system relevance. Biochem Pharmacol. (2021) 191:114463. 10.1016/j.bcp.2021.114463 33577893 PMC7872856

[7] Morin CM Bjorvatn B Chung F Holzinger B Partinen M Penzel T. Insomnia, anxiety, and depression during the COVID-19 pandemic: an international collaborative study. Sleep Med. (2021) 87:38–45. 10.1016/j.sleep.2021.07.035 34508986 PMC8425785

[8] Gualano MR Moro GL Voglino G Bert F Siliquini R. Effects of COVID-19 lockdown on mental health and sleep disturbances in Italy. Int J Environ Res Public Health. (2020) 17:4779. 10.3390/ijerph17134779 32630821 PMC7369943

[9] Pieh C Budimir S Probst T. The effect of age, gender, income, work, and physical activity on mental health during coronavirus disease (COVID-19) lockdown in Austria. J Psychosom Res. (2020) 136:110186. 10.1016/j.jpsychores.2020.110186 32682159 PMC7832650

[10] Casagrande M Favieri F Tambelli R Forte G. The enemy who sealed the world: effects quarantine due to the COVID-19 on sleep quality, anxiety, and psychological distress in the Italian population. Sleep Med. (2020) 75:12–20. 10.1016/j.sleep.2020.05.011 32853913 PMC7215153

[11] Akerstedt T Knutsson A Westerholm P Theorell T Alfredsson L Kecklund G. Sleep disturbances, work stress and work hours: a cross-sectional study. J Psychosom Res. (2002) 53:741–8. 10.1016/S0022-3999(02)00333-1 12217447

[12] Morin CM Jarrin DC Ivers H Mérette C LeBlanc M Savard J. Incidence, persistence, and remission rates of insomnia over 5 years. JAMA Netw Open. (2020) 3:e2018782. Published 2020 Nov 2. 10.1001/jamanetworkopen.2020.18782 33156345 PMC7648256

[13] Seidel S Klösch G Kosheleva A. Help-seeking behavior of young and middle-aged Austrians with chronic insomnia: results from the 2017 national sleep survey. Sleep Epidemiol. (2021) 1:100002. 10.1016/j.sleepe.2021.100002

[14] Chung KF Lee CT Yeung WF Chan MS Chung EW Lin WL. Sleep hygiene education as a treatment of insomnia: a systematic review and meta-analysis. Fam Pract. (2018) 35:365–75. 10.1093/fampra/cmx122 29194467

[15] Riemann D Baum E Cohrs S. S3-Leitlinie Nicht erholsamer Schlaf/Schlafstörungen. Kapitel “Insomnie bei Erwachsenen”. Somnologie. (2017) 21:2–44. 10.1007/s11818-016-0097-x

[16] Buysse DJ Germain A Moul DE Franzen PL Brar LK Fletcher ME. Efficacy of brief behavioral treatment for chronic insomnia in older adults. Arch Intern Med. (2011) 171:887–95. 10.1001/archinternmed.2010.535 21263078 PMC3101289

[17] Crönlein T Lehner A Schüssler P Geisler P Rupprecht R Wetter TC. Changes in subjective-objective sleep discrepancy following inpatient cognitive behavior therapy for insomnia. Behav Ther. (2019) 50:994–1001. 10.1016/j.beth.2019.03.002 31422853

[18] Edinger JD Wohlgemuth WK Radtke RA Coffman CJ Carney CE. Dose-response effects of cognitive-behavioral insomnia therapy: a randomized clinical trial. Sleep. (2007) 30:203–12. 10.1093/sleep/30.2.203 17326546

[19] Bramoweth AD Lederer LG Youk AO Germain A Chinman MJ. Brief behavioral treatment for insomnia vs. cognitive behavioral therapy for insomnia: results of a randomized noninferiority clinical trial among veterans. Behav Ther. (2020) 51:535–47. 10.1016/j.beth.2020.02.002 32586428 PMC10352919

[20] Holzinger B Mayer L Levec K Munzinger M-M Klösch G. Sleep coaching: non-pharmacological treatment of non-restorative sleep in Austrian railway shift workers. Arh Hig Rada Toksikol. (2019) 70:186–93. 10.2478/aiht-2019-70-3244 32597126

[21] Swift N Stewart R Andiappan M Smith A Espie CA Brown JSL. The effectiveness of community day-long CBT-I workshops for participants with insomnia symptoms: a randomised controlled trial. J Sleep Res. (2012) 21:270–80. 10.1111/j.1365-2869.2011.00940.x 21848573

[22] Wong KY Chung KF Au CH. Low-intensity cognitive behavioral therapy for insomnia as the entry of the stepped-care model in the community: a randomized controlled trial. Behav Sleep Med. (2021) 19:378–94. 10.1080/15402002.2020.1764000 32429708

[23] Buysse DJ Reynolds CF 3rd Monk TH Berman SR Kupfer DJ. The pittsburgh sleep quality index: a new instrument for psychiatric practice and research. Psychiatry Res. (1989) 28:193–213. 10.1016/0165-1781(89)90047-4 2748771

[24] Chen PH Kuo HY Chueh KH. Sleep hygiene education: efficacy on sleep quality in working women. J Nurs Res. (2010) 18:283–9. 10.1097/JNR.0b013e3181fbe3fd 21139448

[25] Kakinuma M Takahashi M Kato N Aratake Y Watanabe M Ishikawa Y. Effect of brief sleep hygiene education for workers of an information technology company. Ind Health. (2010) 48:758–65. 10.2486/indhealth.MS1083 20616458

[26] Martin JL Song Y Hughes J Jouldjian S Dzierzewski JM Fung CH. A four-session sleep intervention program improves sleep for older adult day health care participants: results of a randomized controlled trial. Sleep. (2017) 40: zsx0079. 10.1093/sleep/zsx079 28482053 PMC5804980

[27] Krystal AD Edinger JD Wohlgemuth WK Marsh GR. NREM sleep EEG1 frequency spectral correlates of sleep complaints in primary insomnia subtypes. Sleep. (2002) 25:630–40. 10.1093/sleep/25.6.626 12224842

[28] Spiegelhalder K Acker J Baumeister H. Digitale Behandlungsangebote für Insomnie – eine Übersichtsarbeit. Somnologie. (2020) 24:106–14. 10.1007/s11818-020-00238-9

[29] Luik AI Kyle SD Espie CA. Digital cognitive behavioral therapy (dCBT) for insomnia: a state-of-the-science review. Current Sleep Med Rep. (2017) 3:48–56. 10.1007/s40675-017-0065-4 28553574 PMC5427093

[30] Suzuki E Tsuchiya M Hirokawa K Taniguchi T Mitsuhashi T Kawakami N. Evaluation of an internet-based self-help program for better quality of sleep among Japanese workers: a randomized controlled trial. J Occup Health. (2008) 50:387–99. 10.1539/joh.L7154 18716392

[31] Espie CA Emsley R Kyle SD Gordon C Drake CL Siriwardena AN. Effect of digital cognitive behavioral therapy for insomnia on health, psychological well-being, and sleep-related quality of life: a randomized clinical trial. JAMA Psychiatry. (2019) 76:21–30. 10.1001/jamapsychiatry.2018.2745 30264137 PMC6583463

[32] Okajima I Akitomi J Kajiyama I Ishii M Murakami H Yamaguchi M. Effects of a tailored brief behavioral therapy application on insomnia severity and social disabilities among workers with insomnia in japan: a randomized clinical trial. JAMA Net Open. (2020) 3:e202775. 10.1001/jamanetworkopen.2020.2775 32286659 PMC7156995

[33] Ellis JG Cushing T Germain A. Treating acute insomnia: a randomized controlled trial of a “single-shot” of cognitive behavioral therapy for insomnia. Sleep. (2015) 38:971–8. 10.5665/sleep.4752 25515106 PMC4434564

[34] Brinkman JE Reddy V Sharma S. Physiology of Sleep. In: StatPearls Treasure Island (FL): StatPearls Publishing (2021). 29494118

[35] Sadeh A. The role and validity of actigraphy in sleep medicine: an update. Sleep Med Rev. (2011) 15:259–67. 10.1016/j.smrv.2010.10.001 21237680

[36] Marino M Li Y Rueschman MN Winkelman JW Ellenbogen JM Solet JM. Measuring sleep: accuracy, sensitivity, and specifity of wrist actigraphy compared to polysomnography. Sleep. (2013) 36:1747–55. 10.5665/sleep.3142 24179309 PMC3792393

[37] Aili K Åström-Paulsson S Stoetzer U Svartengren M Hillert L. Reliability of actigraphy and subjective sleep measurements in adults: the design of sleep assessments. J Clin Sleep Med. (2017) 13:39–47. 10.5664/jcsm.6384 27707448 PMC5181612

[38] Cole RJ Kripke DF Gruen W Mullaney DJ Gillin JC. Automatic sleep/wake identification from wrist activity. Sleep. (1992) 15:461–9. 10.1093/sleep/15.5.461 1455130

[39] Kristensen TS Hannerz H Høgh A Borg V. The copenhagen psychosocial questionnaire (COPSOQ) - a tool for the assessment and improvement of the psychosocial work environment. Scand J Work Environ Health. (2005) 31:438–49. 10.5271/sjweh.948 16425585

[40] Kovács D Vikoler T. Stress beyond a single dimension? Extending the job-demands resource balance model by a three-dimensional view of stress (Master thesis). University of Salzburg, Institutional Repository at the University of Salzburg Salzburg, Austria. (2018). Available online at: https://eplus.uni-salzburg.at/obvusbhs/download/pdf/5003109?originalFilename=true

[41] Dalbert C. Subjektives Wohlbefinden junger Erwachsener: theoretische und empirische Analysen der Struktur und Stabilität. J Individ Differ. (1992) 13*:* 207–20.

[42] World Health Organization Collaborating Center for Mental Health Psychiatric Research Unit. Denmark: Frederiksborg General Hospital. The WHO-5 Well-Being Index (1998).

[43] Wasserstein RL Schirm AL Lazar NA. Moving to a world beyond “*p* < 005”. Am Stat. (2019) 73:1–19. 10.1080/00031305.2019.1583913

[44] Gavriloff D Sheaves B Juss A Espie CA Miller CB Kyle SD. Sham sleep feedback delivered via actigraphy biases daytime symptom reports in people with insomnia: implications for insomnia disorder and wearable devices. J Sleep Res. (2018) 27:1–10. 10.1111/jsr.12726 29989248

[45] Ho FY Chung KF Yeung WF Ng TH Cheng SK. Weekly brief phone support in self-help cognitive behavioral therapy for insomnia disorder: relevance to adherence and efficacy. Behav Res Ther. (2014) 63:147–56. 10.1016/j.brat.2014.10.002 25461790

[46] van Straten A Cuijpers P. Self-help therapy for insomnia: a meta-analysis. Sleep Med Rev. (2009) 13:61–71. 10.1016/j.smrv.2008.04.006 18952469

[47] Shin J Kim JK. How a good sleep predicts life satisfaction: the role of zero-sum beliefs about happiness. Front Psychol. (2018) 28:1589. 10.3389/fpsyg.2018.01589 30210411 PMC6121950

[48] Steptoe A O'Donnell K Marmot M Wardle J. Positive affect, psychological well-being, and good sleep. J Psychosom Res. (2008) 64:409–15. 10.1016/j.jpsychores.2007.11.008 18374740

[49] Zeitlhofer J Schmeiser-Rieder A Tribl G Rosenberger A Bolitschek J Kapfhammer G. Sleep and quality of life in the Austrian population. Acta Neurol Scand. (2000) 102:249–57. 10.1034/j.1600-0404.2000.102004249.x 11071111

[50] Blume C Hauser T Gruber WR Heib DPJ Winkler T and Schabus M. “How does Austria sleep? “self-reported sleep habits and complaints in an online survey. Sleep Breath. (2020) 24:735–41. 10.1007/s11325-019-01982-5 31838623 PMC7289773

