# Supplementary material for: A low-threshold sleep intervention for improving sleep quality and well-being
Source: Front Psychiatry. 2023 Feb 23;14:1117645. doi: 10.3389/fpsyt.2023.1117645 (PMC9996281; doi:10.3389/fpsyt.2023.1117645)
Supplement: Supplementary file 1 [file Data_Sheet_1.docx]

Supplementary Material

A low-threshold sleep intervention for improving sleep quality and well-being

Esther-Sevil Eigl, Laura Krystin Urban, & Manuel Schabus*

*** Correspondence:**Univ.-Prof. Dr. Manuel Schabus
Hellbrunnerstr. 34
A- 5020 Salzburg, Austria
[manuel.schabus@plus.ac.at](mailto:manuel.schabus@plus.ac.at)

# Supplementary Figures and Tables

## Supplementary Tables

**Table 1**

Content of the daily morning diary

| **Diary** | **Original questionnaire** | **Construct** | **Example** | **Answering mode** |
| --- | --- | --- | --- | --- |
| **Morning Diary** | ‘Gesünder Schlafen’  research group, University of Salzburg, 2018 | Subjective sleep quality  (1 item) | ‘How did you sleep today’ | Sliderbar: 0-100 from ‘very bad’ to ‘very good’ |
|  |  | Vitality  (1 item) | ‘How do you feel at the moment?’ | Sliderbar: 0-100 from ‘faint’ to ‘alive’ |
|  |  | Current mood  (1 item) | ‘How is your mood at the moment?’ | Sliderbar: 0-100 from ‘very bad’ to ‘very good’ |
|  | WHO-Five Well-Being index (World Health Organization, 1998) | Daily well-being  (5 items) | ‘I feel vigorous and active’ or ‘I feel calm and relaxed’ | 5-point Likert scale from ‘I agree’ to ‘I do not agree at all’ |

**Table 2**

Content of the daily evening diary.

| **Diary** | **Original questionnaire** | **Construct** | **Example** | **Answering mode** |
| --- | --- | --- | --- | --- |
| **Evening Diary** | ‘Gesünder Schlafen’ research group, University of Salzburg, 2018 | Evaluation of daily job demands  (1 item) | ‘Today, my job demands made me feel…’ | 5-point Likert scale from ‘very unchallenged’ to ‘very overchallenged’ + ‘I did not work today’ |
|  |  | Evaluation of daily job resources (1 item) | ‘Were there any coping strategies available to you today?’ | 5-point Likert scale from ‘a great many’ to ‘none at all’ + ‘I did not work today’ |
|  | ‘Tension’ Scale of the Perceived Stress Questionnaire (PSQ; Levenstein et al. 1993) | Tension (5 items) | ‘I feel strained’ or  ‘I feel exhausted’ | 5-point Likert scale from ‘I agree’ to ‘I do not agree at all’ |
|  | ‘Recovery Experience Questionnaire’ (REQ; Sonnentag & Fitz, 2007) | Rumination (4 items) | ‘In my leisure time I don’t think about work at all.’ | 5-point Likert scale from ‘I agree’ to ‘I don’t agree at all’ |
|  | ‘Gesünder Schlafen’ research group, University of Salzburg, 2018 | Daytime sleepiness  (1 item) | ‘Were you tired during daytime?’ | Sliderbar: 0-100 from ‘not at all’ to ‘very’ |
|  |  | Vitality  (1 item) | ‘How do you feel at the moment?’ | Sliderbar: 0-100 from ‘faint’ to ‘alive’ |
|  |  | Current mood  (1 item) | ‘How is your mood at the moment?’ | Sliderbar: 0-100 from ‘very bad’ to ‘very good’ |

**Table 3**Trial baseline measures across all groups at pre intervention (T1, T2)

|  | EG1^a^ | | EG2^b^ | | CG^c^ | |
| --- | --- | --- | --- | --- | --- | --- |
| Variable | *M (SD)* | *Mdn* | *M (SD)* | *Mdn* | *M (SD)* | *Mdn* |
| Age | 39.32 (11.02) | 39.00 | 38.21 (11.69) | 34.50 | 41.09 (11.74) | 38.50 |
| PSQI (T1) | 8.50 (3.58) | 8.5 | 7.32 (3.36) | 7.00 | 7.06 (3.14) | 6.00 |
| Objective sleep efficiency | 88.50 (4.33) | 89.57 | 88.80 (4.67) | 88.91 | 88.46 (4.89) | 89.64 |
| Objective sleep onset latency | 7.75 (2.86) | 7.41 | 8.73 (3.90) | 7.40 | 7.27 (2.54) | 6.85 |
| Sleep quality (Morning diary) | 64.17 (11.92) | 70.06 | 68.03 (14.34) | 66.96 | 67.26 (13.84) | 65.81 |
| Well-being (Morning diary) | 3.36 (0.71) | 3.29 | 3.49 (0.56) | 3.40 | 3.43 (0.61) | 3.41 |
| Vitality  (Morning diary) | 57.18 (13.57) | 55.20 | 64.08 (15.40) | 64.50 | 62.24 (16.57) | 61.36 |
| Mood (Morning diary) | 64.74 (12.54) | 65.23 | 70.32 (13.72) | 68.75 | 68.86 (16.07) | 68.93 |

*Note.* Means (*M*), medians (*Mdn*) and standard deviations (*SD*). EG1 = experimental group one, EG2 = experimental group two, CG = control group*. n*^a^=34, *n*^b^= 32 (for objective sleep efficiency and objective sleep onset latency *n*^b^=34), *n*^c^=32

**Table 4**

Change in PSQI Global score from 2019 to 2022 in all three groups.

|  | PSQI 2019 | | PSQI 2022 | |  |  |
| --- | --- | --- | --- | --- | --- | --- |
| Group | *M* | *SD* | *M* | *SD* | *Z*-value | *p*-value |
| EG1^a^ | 9.47 | 4.02 | 4.53 | 3.11 | -2.74 | .006 |
| EG2^b^ | 7.46 | 3.02 | 7.31 | 3.77 | -0.36 | .720 |
| CG^c^ | 6.73 | 2.46 | 6.60 | 2.44 | -0.28 | .776 |

*n*^a^*=15, n*^b^*= 13, n*^c^ *= 15*

**Table 5**

Change in sleep quality from pre to post in participants with PSQI≤10

|  | Sleep quality pre | | Sleep quality post | |  |  |
| --- | --- | --- | --- | --- | --- | --- |
| Group | *M* | *SD* | *M* | *SD* | *t*-value | *p*-value |
| EG1^a^ | 67.56 | 9.29 | 72.50 | 13.93 | -2.20 | .038 |
| EG2^b^ | 70.05 | 13.64 | 73.33 | 13.49 | -1.74 | .093 |
| CG^c^ | 68.60 | 13.65 | 67.45 | 14.79 | 0.54 | .594 |

*n*^a^*=24, n*^b^*= 27, n*^c^ *= 27*

**Figure 1**


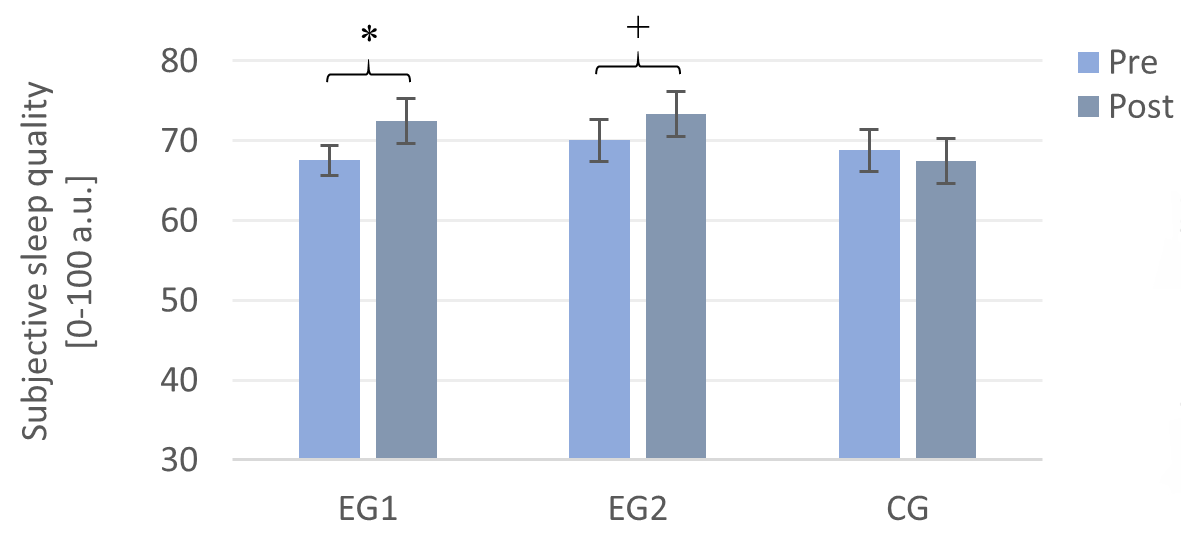


Figure 1. Subjective sleep quality assessed by sleep diaries from pre to post intervention. Note that EG1 improved significantly and EG2 improved tendentially, while CG did not show any improvements. Measurements were assessed daily and averaged over a week each. EG1 (*n* = 343), EG2 (*n* = 32) and CG2 (*n* = 32). Asterisks represent significant results, *p <.05, +p. <.10, two-tailed. Error bars display ±1 standard error. EG1, experimental group one; EG2, experimental group two; CG, control group.

# Additional Analyses

**Wake After Sleep Onset – EG1, EG2, CG**

A 2x3 repeated measures ANOVA with the within-subject factor TIME (Pre, Post), the between-subject factor GROUP (EG1, EG2, CG) and the dependent variable (actigraphy-measured) wake after sleep onset revealed no significant main effect of TIME (*F*(1, 97) = 0.82, *p* = .775, *η²p* = .001), no significant main effect of GROUP (*F*(2, 97) = 0.67, *p* = .512, *η²p* = .014), as well as no significant interaction TIME x GROUP (*F*(2, 97) = 1.96, *p* = .146, *η²p* = .039).

**Total Sleep Time – EG1, EG2, CG**

A 2x3 repeated measures ANVOA with the within-subject factor TIME (Pre, Post), the between-subject factor GROUP (EG1, EG2, CG) and the dependent variable (actigraphy-measured) total sleep time revealed no significant main effect of TIME (*F*(1, 97) = 0.13, *p* = .721, *η²p* = .001), no significant main effect of GROUP (*F*(2, 97) = 1.49, *p* = .231, *η²p* = .030), as well as no significant interaction TIME x GROUP (*F*(2,97) = 0.04, *p* = 963, *η²p* = .001).

**Sleep Intervention vs. control**

**Wake After Sleep onset – EG1+EG2, CG**

A 2x2 repeated measures ANOVA with the within-subject factor TIME (Pre, Post), the between-subject factor GROUP (EG1+EG2, CG) and the dependent variable (actigraphy-measured) wake after sleep onset revealed no significant main effect of TIME (*F*(1, 98) = 0.87, *p* = .354, *η²p* = .009), nor for GROUP (*F*(1, 98) = 0.94, *p* = .334, *η²p* = .010) yet a significant interaction TIME x GROUP (*F*(1,98) = 3.96, *p* = .049, *η²p* = .039). According to post-hoc tests there was no significant change from pre- to post-intervention neither in the ‘sleep intervention group’ (Pre: *M* = 46.58, Post: *M* = 45.06; *t*(67) = 1.20, *p* = .233, nor in the CG (Pre: *M* = 48.44, Post: *M* = 52.64 ; *t*(31) = -1.30, *p* = .203).

**Total Sleep Time – EG1+EG2, CG**

A 2x2 repeated measures ANOVA with the within-subject factor TIME (Pre, Post), the between-subject factor GROUP (EG1+EG2, CG) and the dependent variable (actigraphy-measured) total sleep time revealed no significant main effect of TIME (*F*(1, 98)= 0.17, *p* = .685, *η²p* = .002), nor for GROUP (*F*(1, 98) = 1.08, *p* = .302, *η²p* = .011), as well as no significant interaction TIME x GROUP (*F*(1, 98)= 0.05, *p* = 832, *η²p* = .000).
